# Supplementary material for: Plant roots affect free-living diazotroph communities in temperate grassland soils despite decades of fertilization
Source: Commun Biol. 2024 Jul 11;7:846. doi: 10.1038/s42003-024-06522-w (PMC11237082; doi:10.1038/s42003-024-06522-w)
Supplement: Supplementary file 2 — Supplementary Information [file 42003_2024_6522_MOESM2_ESM.pdf]

SUPPLEMENTARY INFORMATION

Plant roots affect free-living diazotroph communities in temperate grassland soils despite decades of fertilization

Supplementary Figure 1 Taxonomic composition of the diazotroph community in microenvironments associated with grasses and herbs across investigated fertilization treatments.

Supplementary Figure 2 Species richness of diazotroph communities in microenvironments associated with grasses and herbs.

Supplementary Figure 3 Diazotroph abundance in bulk soil and rhizosphere soil associated with four investigated plant species among different fertilization treatments.

Supplementary Figure 4 Species richness of diazotroph communities and general microbial communities in bulk soils across different investigated fertilization treatments.

Supplementary Figure 5 Diazotroph and general community composition in bulk soils among different years.

Supplementary Table 1 Edaphic properties for investigate field treatments

Supplementary Table 2 Effects of plant type and plant species on the diazotroph and general microbial community composition.

Supplementary Table 3 Effects of pairwise comparisons of plant-associated compartments of individual grasses and herbs on the diazotroph and general microbial community.

Supplementary Table 4 Effects of pairwise microenvironment comparisons of grasses and herbs on the diazotroph and general microbial community.

Supplementary Table 5 Effects of pairwise comparisons of microenvironments between grasses and herbs on the diazotroph and general microbial community.

Supplementary Table 6 Effect of fertilization and plant species on the diazotroph and general microbial community composition.

Supplementary Table 7 Effect of fertilization and plant species on the diazotroph and general microbial community composition excluding sampled from the NPK treatment.

Supplementary Table 8 Number of investigated samples.

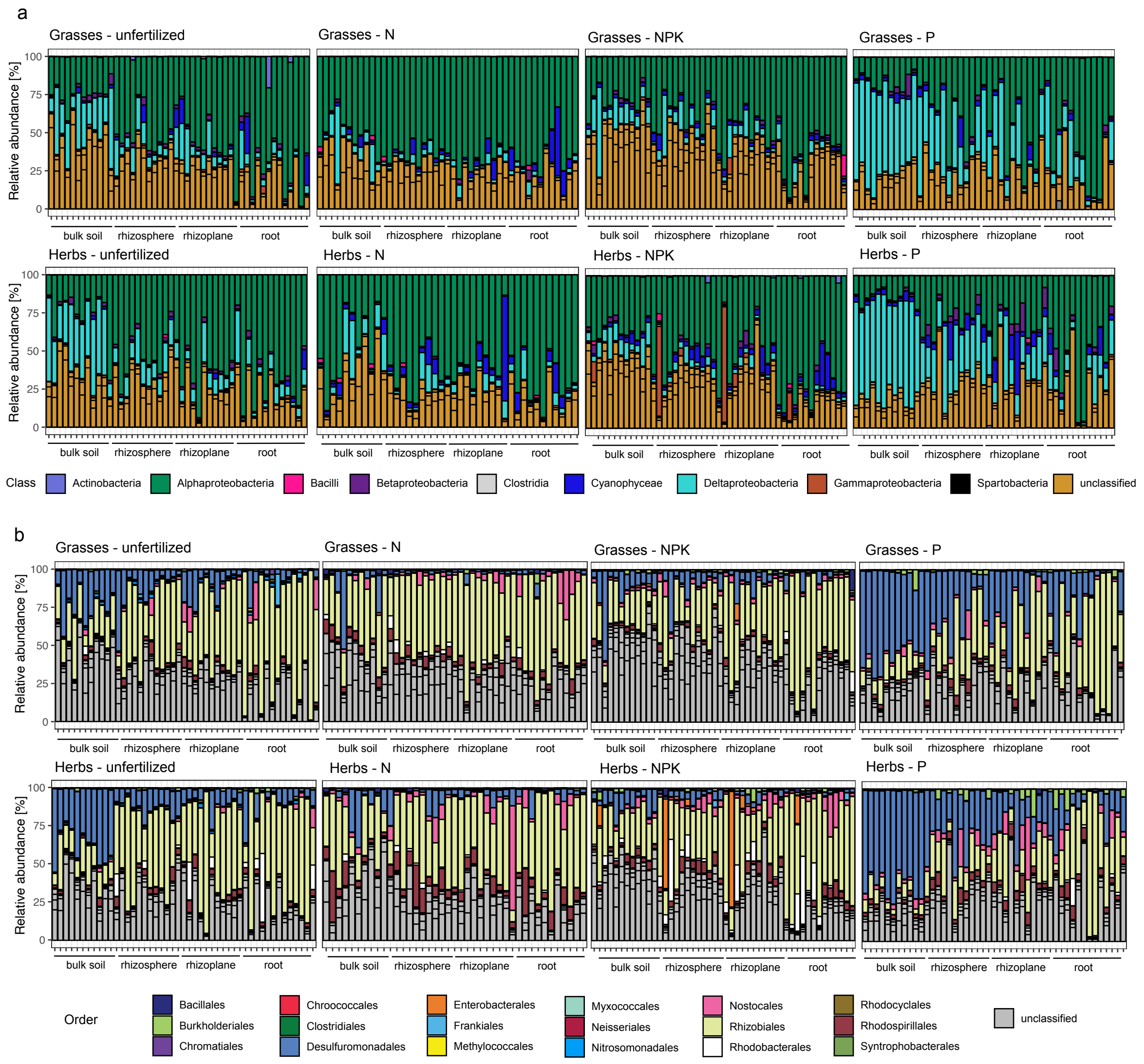

Supplementary Figure 1

Taxonomic composition of the diazotroph community in microenvironments (bulk soil, rhizosphere, rhizoplane, root) associated with grasses and herbs across investigated fertilization treatments. Stacked bars reflect relative abundances [%] and are colored based on (a) class level and (b) order level.

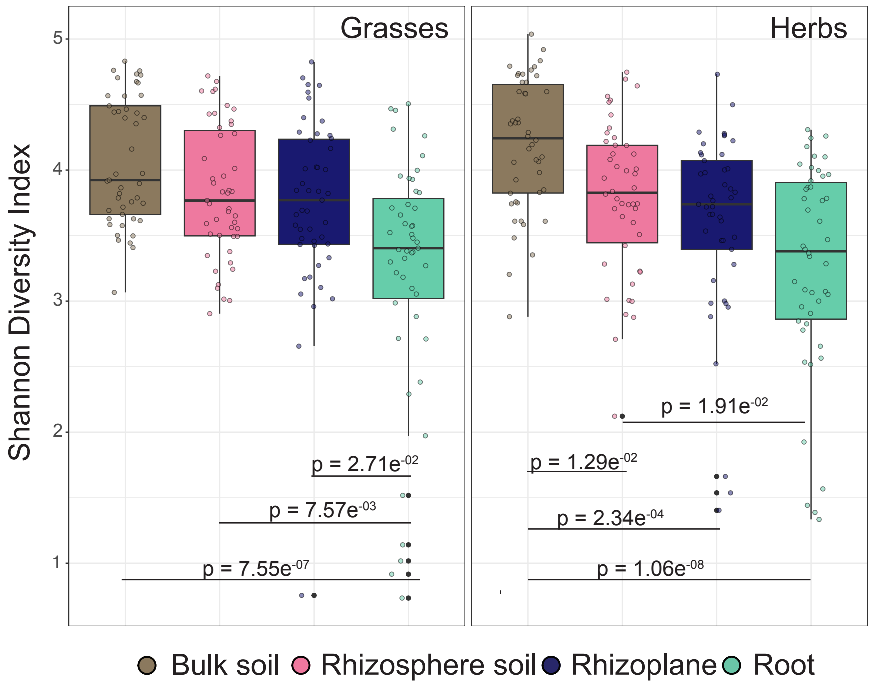

Supplementary Figure 2

Species richness of diazotroph communities in bulk soil (brown), rhizosphere soil (pink), rhizoplane (blue) and root (turquoise) microenvironments associated with grasses and herbs based on Shannon diversity index. P -values indicate significant differences across microenvironments based on Kruskal Wallis - Dunn test. Lower and upper hinges represent the first and third quartiles.

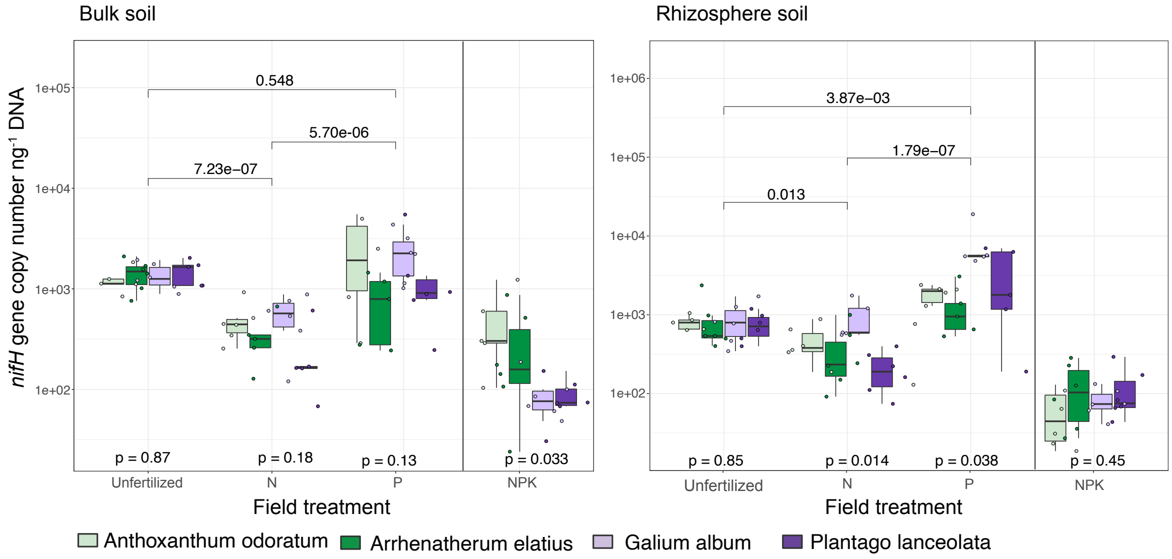

Supplementary Figure 3

Diazotroph abundance in bulk soil and rhizosphere soil associated with four investigated plant species among different field treatments. Unfertilized, N and P field treatments were sampled in 2014, NPK treatment was sampled in 2018. P-values depict significant and non-significant differences among plant species (Mann-Whitney U test) and field treatments (Kruskal-Wallis, Dunn test; pooled data across all plant species were used per field treatment and p-values adjusted to Benjamini-Hochberg corrections for multiple testing are depicted). Lower and upper hinges represent the first and third quartiles.

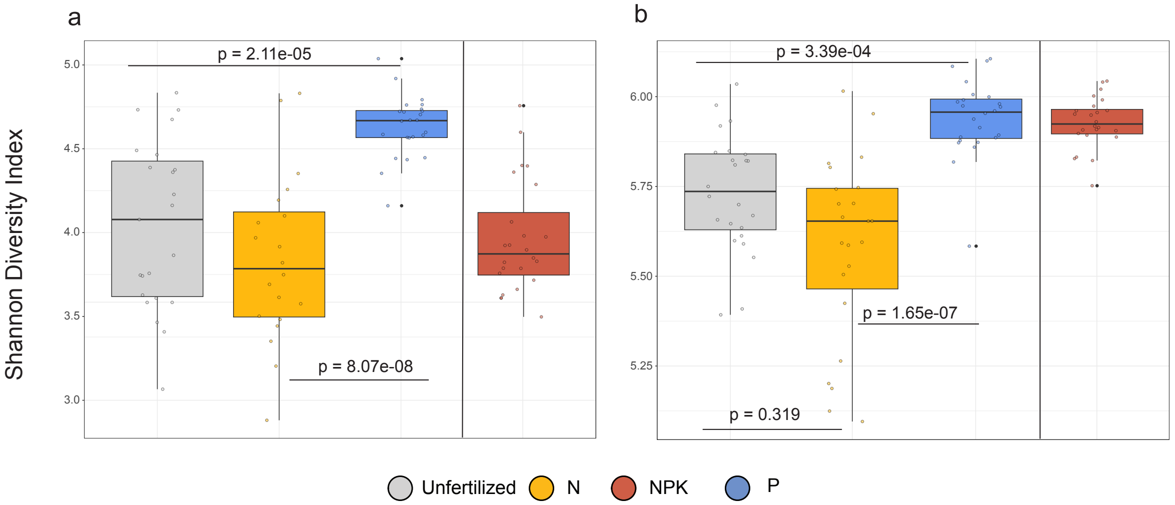

Supplementary Figure 4

Species richness of (a) diazotroph communities and (b) general microbial communities in bulk soils across different investigated field treatment (unfertilized – grey; N – yellow, NPK – red; P – blue) based on Shannon diversity index. Unfertilized, N and P field treatments were sampled in 2014, NPK treatment was sampled in 2018. P -values indicate significant differences across field treatments based on (a) ANOVA – TukeyHSD test and (b) Kruskal Wallis - Dunn test (p-values adjusted to to Benjamini-Hochberg corrections for multiple testing). Lower and upper hinges represent the first and third quartiles.

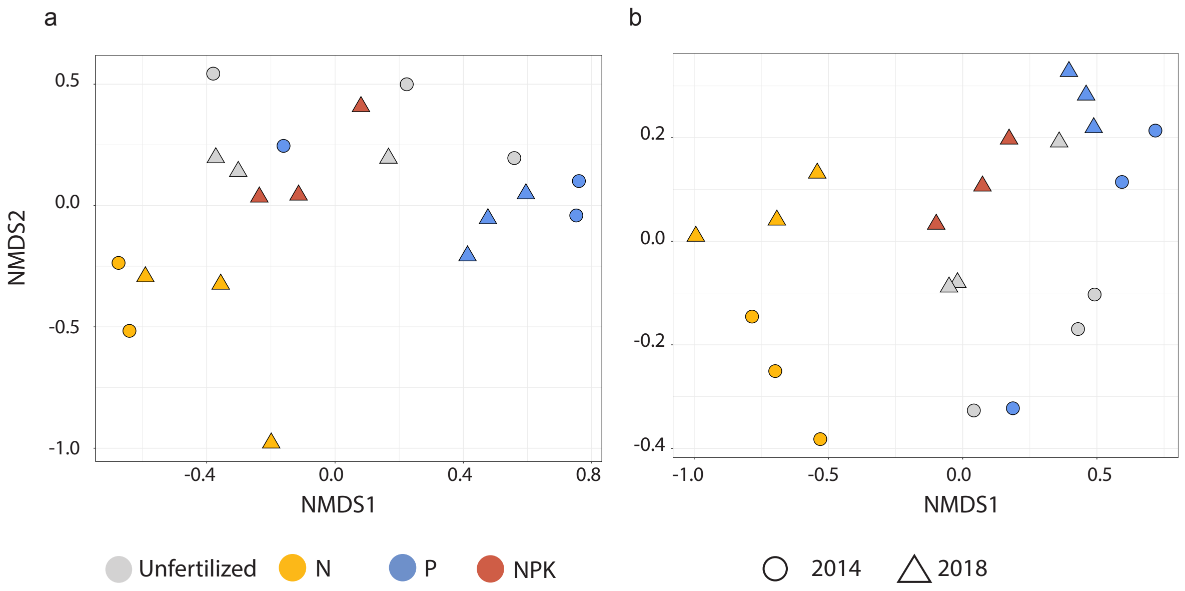

Supplementary Figure 5

Community composition in bulk soils from 2014 and 2018. Non-metric multidimensional scaling (NMDS) ordination plots of (a) diazotroph (stress value = 8.50) and (b) general microbial (stress value = 5.17) communities based on Bray-Curtis metric, illustrating beta-diversity of bulk soil samples sampled from unfertilized (grey), N fertilized (yellow) and P fertilized (blue) plots among sampling years 2014 (circle) and 2018 (triangle). Soil from NPK fertilization (red) was only sampled in 2018.

Our study started with samples taken in 2014 of plots "unfertilized", "N addition" and "P addition". Based on these data, we wanted to include a fully fertilized plot – NPK. As such, the NPK data set was sampled in 2018. To avoid repeated major disturbances in the plots already sampled in 2014 (caused by taking out cores of ~20 cm diameter containing the roots system of grasses and herbs), it was not possible to re-sample all plants across all treatments in 2018. However, we were able to resample bulk soil to determine if there was any change in the microbial community over a 4-year time span.

We used these available bulk soil samples from 2014 and 2018 to investigate any changes in the community due to time. Our analysis demonstrates that the diazotroph and general microbial community composition in bulk soils did not significantly change in the diazotrophic (Fig. S5, panel a) nor general microbial community (Fig. S5, panel b) across the investigated time period (2014-2018). Specifically, no significant changes were detected in the diazotroph and general microbial community composition based on PERMANOVA analysis (below) in the N, P and unfertilized treatments across the investigated years.

For statistical analysis where we directly combined data from all treatments, we repeated the analysis excluding the NPK treatment to rule out that the observed effects stemmed from different sampling time points. The results indicated that the observed effects of fertilization treatments (N,P,unfertilized alone) were virtually identical to the observed effects when including the NPK treatment in the analysis (Supplementary Table 7).

(a) PERMANOVA results comparing the diazotroph community composition between years (p-values adjusted to Benjamini-Hochberg corrections for multiple testing are displayed). Additionally, the diazotroph community composition across fertilization treatments sampled in 2018 was compared.

N treatment (comparing 2014 & 2018) : R^2 = 0.236, p = 0.4;
P treatment (comparing 2014 & 2018): R^2 = 0.223, p = 0.3;
unfertilized treatment (comparing 2014, 2018): R^2 = 0.217, p = 0.5
comparing all treatments (2018): R^2= 0.54, p = 7e-04

(b) PERMANOVA results comparing the general microbial community composition among years (p-values adjusted to Benjamini-Hochberg corrections for multiple testing are displayed). Additionally, the general microbial community composition across fertilization treatments sampled in 2018 was compared.
N treatment (comparing 2014 & 2018) : R^2 = 0.259, p = 0.2;
P treatment (comparing 2014 & 2018): R^2 = 0.233, p = 0.1;
unfertilized treatment (comparing 2014 & 2018): R^2 = 0.249, p = 0.2
comparing all treatments (2018): R^2=0.566, p = 8e-04

As such, the differences in sampling times did not significantly affect the observed results. Therefore, we felt confident to combine the data from 2014 and 2018 to explore the effect fertilization, plant species and microenvironments had in shaping the diazotrophic community in these grasslands.

Supplementary Table 1

Edaphic properties of investigated field treatments including (a) soil parameters and (b) plant growth data.

a) Soil parameters for all investigated field treatments. Soil sampled in October 2011, 2016 and 2021 (approximately 4 months after N fertilization) was analyzed. Presented data include mean and standard error (SE) calculations across the three years (n=3).

	Field treatment							
	Unfertilized		N		P		NPK	
Soil parameters	Mean (n=3)	SE	Mean (n=3)	SE	Mean (n=3)	SE	Mean (n=3)	SE
pH	5.46	0.07	4.41	0.10	6.00	0.06	5.43	0.10
Total N [%]	0.39	0.02	0.48	0.06	0.36	0.01	0.37	0.02
C organic [%]	4.83	0.56	5.16	0.66	4.26	0.28	4.11	0.51
C:N	12.18	0.97	10.81	0.63	11.97	0.40	11.12	0.77
P [mg kg $^{-1}$]	20.67	1.20	24.33	2.85	119.67	4.91	27.67	0.88
K [mg kg $^{-1}$]	79.00	12.50	71.67	13.92	68.67	11.22	154.67	23.70
Fe [mg kg $^{-1}$]	315.40	11.70	510.93	56.91	540.60	22.33	344.90	15.00

b) Plant growth data for all investigated field treatments. Data were obtained three times a year (end of May/early June, July, September) in 2011, 2016 and 2021. Presented data include mean and standard error (SE) calculations of three replicate plot and three timepoints across the three years (n=27).

	Field treatment							
	Unfertilized		N		P		NPK	
Plant yield	Mean (n=27)	SE	Mean (n=27)	SE	Mean (n=27)	SE	Mean (n=27)	SE
Yield (Dry Weight, dt ha^{-1})	6.03	0.60	7.65	0.73	8.21	0.55	25.71	1.46
Coverage [%]	94.30	1.16	93.70	0.90	94.93	1.02	96.59	0.45
Grasses [weight %]	64.96	3.56	86.68	1.23	53.41	2.64	71.11	4.08
Leguminoses [weight%]	6.59	1.11	1.51	0.20	6.67	0.69	3.41	0.35
Herbs [weight %]	28.44	2.89	11.81	1.14	39.93	2.40	25.48	3.86

Supplementary Table 2

Effects of group of plant species (plant type) and individual plant species on the diazotroph and general microbial community composition associated with rhizosphere, rhizoplane and root compartments in the four investigated fertilization treatments based on PERMANOVA on the Bray-Curtis dissimilarity index. Significant effects are shown in bold.

				Diazotroph community					General microbial community				
Plant	Plant-associated compartment	Fertilization	Factors	Df	SumsOfSqs	R^2	F	p-value	Df	SumsOfSqs	R^2	F	p-value
all	rhizosphere, rhizoplane, root	all	Plant Type	1	0.825	0.011	3.05	**0.0007**	1	1.764	0.028	7.97	**0.0001**
			Residuals	276	75.712	0.989			276	61.133	0.972		
			Total	277	75.537	1.000			277	62.898	1.000		
		unfertilized	Plant Type	1	0.541	0.032	2.28	**0.0091**	1	0.683	0.050	3.67	**0.0001**
			Residuals	70		0.968			70	13.011	0.950		
			Total	71	17.157	1.000			71	13.694	1.000		
		N	Plant Type	1	0.705	0.054	3.67	**0.0001**	1	1.188	0.083	5.63	**0.0001**
			Residuals	64	12.294	0.946			62	13.084	0.917		
			Total	65	12.999	1.000			63	14.272	1.000		
		NPK	Plant Type	1	0.524	0.034	2.43	**0.0115**	1	0.562	0.043	3.12	**0.0003**
			Residuals	70	15.122	0.967			70	12.613	0.957		
			Total	71	15.646	1.000			71	13.175	1.000		
		P	Plant Type	1	0.593	0.043	2.93	**0.0006**	1	0.830	0.061	4.44	**0.0001**
			Residuals	66	13.353	0.957			68	12.720	0.939		
			Total	67	13.945	1.000			69	13.550	1.000		
grasses	rhizosphere, rhizoplane, root	all	Plant Species	1	0.566	0.015	2.06	**0.0158**	1	0.699	0.026	3.77	**0.0002**
			Residuals	139	38.311	0.985			140	25.997	0.974		
			Total	140	38.877	1.000			141	26.697	1.000		
		unfertilized	Plant Species	1	0.328	0.037	1.31	0.1909	1	0.386	0.070	2.55	**0.0086**
			Residuals	34	8.504	0.963			34	5.136	0.930		
			Total	35	8.832	1.000			35	5.522	1.000		
		N	Plant Species	1	0.331	0.049	1.75	**0.0473**	1	0.445	0.067	2.43	**0.0114**
			Residuals	34	6.441	0.951			34	6.212	0.933		
			Total	35	6.772	1.000			35	6.657	1.000		
		NPK	Plant Species	1	0.414	0.058	2.09	**0.0321**	1	0.190	0.049	1.75	0.0667
			Residuals	34	6.739	0.942			34	3.704	0.951		
			Total	35	7.153	1.000			35	3.895	1.000		
		P	Plant Species	1	0.345	0.050	1.64	0.0662	1	0.405	0.076	2.65	**0.0046**
			Residuals	31	6.536	0.950			32	4.900	0.924		
			Total	32	6.882	1.000			33	5.305	1.000		
herbs	rhizosphere, rhizoplane, root	all	Plant Species	1	0.587	0.016	2.25	**0.0106**	1	0.820	0.029	4.20	**0.0001**
			Residuals	135	35.248	0.984			139	27.127	0.971		
			Total	136	35.835	1.000			140	27.947	1.000		
		unfertilized	Plant Species	1	0.451	0.058	2.09	**0.028**	1	0.360	0.072	2.63	**0.0022**
			Residuals	34	7.333	0.942			34	4.651	0.928		
			Total	35	7.783	1.000			35	5.010	1.000		
		N	Plant Species	1	0.447	0.081	2.47	**0.0082**	1	0.591	0.099	3.40	**0.001**
			Residuals	28	5.075	0.919			31	5.388	0.901		
			Total	29	5.522	1.000			32	5.979	1.000		
		NPK	Plant Species	1	0.723	0.091	3.39	**0.0008**	1	0.392	0.082	3.02	**0.0052**
			Residuals	34	7.246	0.909			34	4.410	0.918		
			Total	35	7.969	1.000			35	4.801	1.000		
		P	Plant Species	1	0.320	0.049	1.71	0.0539	1	0.259	0.066	2.39	**0.0053**
			Residuals	33	6.152	0.951			34	3.674	0.934		
			Total	34	6.471	1.000			35	3.933	1.000		

Supplementary Table 3

Effects of plant-associated compartments (rs=rhizosphere, rp=rhizoplane) of individual grasses (Anth. = *Anthoxanthum odoratum*, Arrhen. = *Arrhenatherum elatius*) and herbs (Gal. = *Galium album*, Plant. = *Plantago lanceolata*) on the diazotroph and general microbial community based on pairwise comparisons using the Bray-Curtis dissimilarity index. Significant effects are shown in bold based on an adjusted p-value due to multiple comparisons using the Benjamini-Hochberg procedure (BH).

			Diazotroph community						General microbial community					
Plants	Fertilization	Pairs	Df	SumsOfSqs	F	R^2	p-value	p-adj. (BH)	Df	SumsOfSqs	F	R^2	p-value	p-adj. (BH)
grasses	unfertilized	Anth. - rs vs Arrhen.- rs	1	0.128	0.702	0.066	0.729	0.8727	1	0.145	0.997	0.091	0.378	0.4550
		Arrhen.- rp vs Anth. - rp	1	0.212	0.914	0.084	0.479	0.7987	1	0.182	1.314	0.116	0.186	0.2536
		Arrhen.- root vs Anth. - root	1	0.177	0.547	0.052	0.949	0.9494	1	0.192	1.474	0.128	0.083	0.1250
	N	Arrhen.- rs vs Anth. - rs	1	0.197	1.281	0.114	0.204	0.3543	1	0.179	0.981	0.089	0.401	0.5010
		Arrhen.- rp vs Anth. - rp	1	0.163	0.985	0.090	0.474	0.6466	1	0.166	0.904	0.083	0.498	0.5746
		Arrhen.- root vs Anth. - root	1	0.169	0.768	0.071	0.795	0.8521	1	0.308	2.580	0.205	0.005	**0.0084**
	NPK	Arrhen.- rs vs Anth. - rs	1	0.141	0.898	0.082	0.415	0.5183	1	0.086	1.004	0.091	0.409	0.4606
		Arrhen.- rp vs Anth. - rp	1	0.137	0.750	0.070	0.668	0.7669	1	0.082	0.956	0.087	0.430	0.4606
		Arrhen.- root vs Anth. - root	1	0.280	1.266	0.112	0.215	0.3462	1	0.114	1.072	0.097	0.327	0.4455
	P	Arrhen.- rs vs Anth. - rs	1	0.130	0.767	0.079	0.741	0.8552	1	0.177	1.381	0.133	0.164	0.2239
		Anth. - rp vs Arrhen.- rp	1	0.242	1.139	0.112	0.355	0.6223	1	0.254	2.248	0.200	0.030	0.0500
		Anth. - root vs Arrhen.- root	1	0.340	1.376	0.133	0.161	0.4836	1	0.188	1.158	0.104	0.283	0.3136
herbs	unfertilized	Gal.- rs vs Plant.- rs	1	0.117	0.663	0.062	0.753	0.8708	1	0.073	0.606	0.057	0.868	0.8677
		Gal.- rp vs Plant.- rp	1	0.186	0.843	0.078	0.561	0.8408	1	0.136	1.322	0.117	0.190	0.2588
		Plant.- root vs Gal.- root	1	0.282	1.059	0.096	0.370	0.7901	1	0.500	3.676	0.269	0.002	**0.0060**
	N	Gal.- rs vs Plant.- rs	1	0.241	1.446	0.153	0.112	0.5261	1	0.192	1.202	0.118	0.230	0.2654
		Gal.- rp vs Plant.- rp	1	0.284	1.398	0.166	0.088	0.5261	1	0.257	1.560	0.148	0.109	0.1488
		Gal.- root vs Plant.- root	1	0.119	0.586	0.061	0.897	0.9434	1	0.571	3.972	0.306	0.003	**0.0140**
	NPK	Gal.- rs vs Plant.- rs	1	0.224	1.275	0.113	0.227	0.3782	1	0.153	2.089	0.173	0.035	**0.0406**
		Gal.- rp vs Plant.- rp	1	0.245	1.004	0.091	0.447	0.6091	1	0.138	1.550	0.134	0.089	0.0949
		Gal.- root vs Plant.- root	1	0.428	1.840	0.155	0.057	0.2126	1	0.552	5.408	0.351	0.002	**0.0047**
	P	Gal.- rs vs Plant.- rs	1	0.101	0.686	0.064	0.884	0.9366	1	0.068	0.900	0.083	0.543	0.5425
		Gal.- rp vs Plant.- rp	1	0.149	0.810	0.075	0.676	0.9217	1	0.096	1.223	0.109	0.121	0.1393
		Gal.- root vs Plant.- root	1	0.229	1.003	0.100	0.422	0.9217	1	0.463	5.263	0.345	0.003	**0.0047**

Supplementary Table 4

Effects of microenvironment (bs=bulk soil, rs=rhizosphere, rp=rhizoplane) of grasses and herbs on the diazotroph and the general microbial community based on pairwise comparisons using the Bray-Curtis dissimilarity index. Significant effects are shown in bold based on an adjusted p-value due to multiple comparisons using the Benjamini-Hochberg procedure (BH).

			Diazotroph community						General microbial community					
Plants	Fertilization	Pairs	Df	SumsOfSqs	F	R^2	p-value	p-adj. (BH)	Df	SumsOfSqs	F	R^2	p-value	p-adj. (BH)
grasses	unfertilized	bs vs rs	1	0.776	4.397	0.167	0.0002	**0.0004**	1	0.776	4.397	0.167	0.0002	**0.0004**
		bs vs rp	1	0.871	4.294	0.163	0.0001	**0.0003**	1	0.871	4.294	0.163	0.0002	**0.0004**
		bs vs root	1	1.485	6.114	0.217	0.0001	**0.0003**	1	1.485	6.114	0.217	0.0001	**0.0004**
		rs vs root	1	0.810	3.325	0.131	0.0008	**0.0012**	1	0.810	3.325	0.131	0.0012	**0.0018**
		rs vs rp	1	0.196	0.962	0.042	0.4492	0.4492	1	0.196	0.962	0.042	0.4374	0.4374
		root vs rp	1	0.403	1.492	0.063	0.1241	0.1489	1	0.403	1.492	0.063	0.1224	0.1469
	N	bs vs rs	1	0.337	1.944	0.093	0.0365	**0.0438**	1	0.337	1.944	0.093	0.0362	**0.0434**
		bs vs rp	1	0.518	2.911	0.133	0.0004	**0.0008**	1	0.518	2.911	0.133	0.0002	**0.0004**
		bs vs root	1	0.960	4.645	0.196	0.0001	**0.0006**	1	0.960	4.645	0.196	0.0001	**0.0003**
		rs vs root	1	0.758	4.064	0.156	0.0002	**0.0006**	1	0.758	4.064	0.156	0.0001	**0.0003**
		rs vs rp	1	0.168	1.041	0.045	0.3964	0.3964	1	0.168	1.041	0.045	0.3912	0.3912
		root vs rp	1	0.342	1.797	0.076	0.0323	**0.0438**	1	0.342	1.797	0.076	0.0325	**0.0434**
	NPK	bs vs rs	1	0.224	1.389	0.059	0.1766	0.2119	1	0.224	1.389	0.059	0.1812	0.2174
		bs vs rp	1	0.381	2.201	0.091	0.0463	0.0695	1	0.381	2.201	0.091	0.0429	0.0644
		bs vs root	1	1.036	5.256	0.193	0.0001	**0.0006**	1	1.036	5.256	0.193	0.0002	**0.0006**
		rs vs root	1	0.847	4.433	0.168	0.0002	**0.0006**	1	0.847	4.433	0.168	0.0001	**0.0006**
		rs vs rp	1	0.165	0.985	0.043	0.3977	0.3977	1	0.165	0.985	0.043	0.4001	0.4001
		root vs rp	1	0.462	2.279	0.094	0.0201	**0.0402**	1	0.462	2.279	0.094	0.02	**0.0400**
	P	bs vs rs	1	0.389	2.665	0.113	0.0008	**0.0016**	1	0.389	2.665	0.113	0.0007	**0.0016**
		bs vs rp	1	0.550	3.240	0.134	0.0004	**0.0012**	1	0.550	3.240	0.134	0.0008	**0.0016**
		bs vs root	1	1.105	5.827	0.217	0.0001	**0.0006**	1	1.105	5.827	0.217	0.0001	**0.0006**
		rs vs root	1	0.392	1.859	0.085	0.0317	**0.0476**	1	0.392	1.859	0.085	0.0285	**0.0428**
		rs vs rp	1	0.120	0.629	0.030	0.8284	0.8284	1	0.120	0.629	0.030	0.8295	0.8295
		root vs rp	1	0.254	1.075	0.051	0.3518	0.4222	1	0.254	1.075	0.051	0.3566	0.4279
herbs	unfertilized	bs vs rs	1	0.792	4.784	0.186	0.0003	**0.0006**	1	0.401	3.097	0.123	0.0001	**0.0001**
		bs vs rp	1	0.963	5.069	0.194	0.0001	**0.0003**	1	0.510	3.832	0.148	0.0001	**0.0001**
		bs vs root	1	1.303	6.020	0.223	0.0001	**0.0003**	1	0.971	6.467	0.227	0.0001	**0.0001**
		rs vs root	1	0.478	2.178	0.090	0.0174	**0.0261**	1	0.576	3.805	0.147	0.0001	**0.0001**
		rs vs rp	1	0.172	0.888	0.039	0.5084	0.6101	1	0.108	0.800	0.035	0.8054	0.8054
		root vs rp	1	0.199	0.818	0.036	0.6254	0.6254	1	0.499	3.220	0.128	0.0001	**0.0001**
	N	bs vs rs	1	0.318	1.618	0.078	0.0788	0.1730	1	0.577	3.467	0.169	0.0006	**0.0012**
		bs vs rp	1	0.319	1.486	0.076	0.0865	0.1730	1	0.562	2.997	0.136	0.0022	**0.0033**
		bs vs root	1	0.613	2.988	0.130	0.0018	**0.0108**	1	1.020	5.310	0.218	0.0001	**0.0006**
		rs vs root	1	0.207	1.117	0.056	0.3224	0.4836	1	0.525	2.992	0.158	0.0004	**0.0012**
		rs vs rp	1	0.095	0.493	0.028	0.9764	0.9764	1	0.177	1.042	0.061	0.3321	0.3321
		root vs rp	1	0.137	0.674	0.036	0.8067	0.9680	1	0.453	2.304	0.113	0.004	**0.0048**
	NPK	bs vs rs	1	0.273	1.698	0.072	0.0959	0.1439	1	0.278	2.175	0.090	0.0009	**0.0011**
		bs vs rp	1	0.531	2.752	0.111	0.0059	**0.0177**	1	0.430	3.034	0.121	0.0001	**0.0002**
		bs vs root	1	1.049	5.341	0.195	0.0001	**0.0006**	1	1.348	8.497	0.279	0.0001	**0.0002**
		rs vs root	1	0.467	2.169	0.090	0.0261	0.0522	1	1.117	7.082	0.244	0.0001	**0.0002**
		rs vs rp	1	0.149	0.703	0.031	0.7256	0.7256	1	0.184	1.303	0.056	0.0836	0.0836
		root vs rp	1	0.214	0.866	0.038	0.558	0.6696	1	0.841	4.898	0.182	0.0001	**0.0002**
	P	bs vs rs	1	0.639	5.248	0.193	0.0002	**0.0004**	1	0.429	3.576	0.140	0.0001	**0.0001**
		bs vs rp	1	1.018	7.231	0.247	0.0001	**0.0003**	1	0.625	4.931	0.183	0.0001	**0.0001**
		bs vs root	1	1.523	9.419	0.310	0.0001	**0.0003**	1	1.372	9.413	0.300	0.0001	**0.0001**
		rs vs root	1	0.484	2.635	0.111	0.0061	**0.0092**	1	0.841	5.403	0.197	0.0001	**0.0001**
		rs vs rp	1	0.138	0.855	0.037	0.6467	0.6467	1	0.169	1.233	0.053	0.0672	0.0672
		root vs rp	1	0.319	1.567	0.069	0.0946	0.1135	1	0.704	4.335	0.165	0.0001	**0.0001**

Supplementary Table 5

Effects of microenvironments between grasses and herbs (bs=bulk soil, rs=rhizosphere, rp=rhizoplane) on the diazotroph and the general microbial community using pairwise comparisons based on the Bray-Curtis dissimilarity index. Significant effects are shown in bold based on an adjusted p-value due to multiple comparisons using the Benjamini-Hochberg procedure (BH).

			**Diazotroph community**						**General microbial community**					
Plants	Fertilization	Pairs	Df	SumsOfSqs	F	R^2	p-value	p-adj. (BH)	Df	SumsOfSqs	F	R^2	p-value	p-adj. (BH)
all	unfertilized	grass bs vs herb bs	1	0.188	1.119	0.051	0.2808	0.3276	1	0.191	1.274	0.055	0.1354	0.1458
		grass rs vs herb rs	1	0.097	0.557	0.025	0.8925	0.8925	1	0.306	1.919	0.080	0.0072	**0.0084**
		grass rp vs herb rp	1	0.257	1.148	0.050	0.2693	0.3276	1	0.274	1.654	0.070	0.021	**0.0235**
		grass root vs herb root	1	0.448	1.552	0.066	0.0676	0.0996	1	0.564	3.080	0.123	0.0001	**0.0001**
	N	grass bs vs herb bs	1	0.287	1.391	0.072	0.1375	0.1674	1	0.404	2.038	0.088	0.0249	**0.0268**
		grass rs vs herb rs	1	0.225	1.362	0.064	0.1519	0.1772	1	0.523	2.915	0.139	0.0005	**0.0007**
		grass rp vs herb rp	1	0.297	1.598	0.078	0.0292	**0.0481**	1	0.444	2.213	0.100	0.0033	**0.0037**
		grass root vs herb root	1	0.512	2.491	0.106	0.0023	**0.0059**	1	0.921	5.099	0.203	0.0001	**0.0002**
	NPK	grass bs vs herb bs	1	0.100	0.644	0.028	0.7140	0.7289	1	0.118	0.896	0.039	0.5637	0.5637
		grass rs vs herb rs	1	0.244	1.459	0.062	0.1344	0.1981	1	0.236	1.750	0.074	0.0146	**0.0186**
		grass rp vs herb rp	1	0.301	1.528	0.068	0.0661	0.0841	1	0.232	1.546	0.066	0.0173	**0.0211**
		grass root vs herb root	1	0.288	1.188	0.056	0.2494	0.2793	1	0.566	3.291	0.130	0.0001	**0.0002**
	P	grass bs vs herb bs	1	0.195	1.700	0.072	0.0217	**0.0357**	1	0.273	1.888	0.079	0.0001	**0.0001**
		grass rs vs herb rs	1	0.210	1.370	0.061	0.1068	0.1246	1	0.260	1.810	0.079	0.0001	**0.0001**
		grass rp vs herb rp	1	0.301	1.528	0.068	0.0661	0.0841	1	0.295	1.857	0.081	0.0001	**0.0001**
		grass root vs herb root	1	0.288	1.188	0.056	0.2494	0.2793	1	0.829	4.514	0.170	0.0001	**0.0001**

Supplementary Table 6

Effect of fertilization and plant species on the diazotroph and the general microbial community composition based on PERMANOVA using the Bray-Curtis dissimilarity index. Significant effects are shown in bold.

				Diazotroph community					General microbial community				
Plants	Fertilization	Microenvironment	Factors	Df	SumsOfSqs	R^2	F	p-value	Df	SumsOfSqs	R^2	F	p-value
all	all	bulk soil	Treatment	3	8.482	0.378	17.761	**0.0001**	3	5.757	0.284	12.602	**0.0001**
			Plant Species	3	0.565	0.025	1.183	0.2268	3	0.765	0.038	1.676	**0.009**
			Treatment:Plant Species	9	1.438	0.064	1.004	0.4455	9	1.726	0.085	1.259	**0.0347**
			Residuals	75	11.939	0.532			79	12.030	0.593		
			Total	90	22.423	1.000			94	20.278	1.000		
		rhizosphere soil	Treatment	3	6.825	0.315	13.722	**0.0001**	3	3.497	0.199	7.705	**0.0001**
			Plant Species	3	0.732	0.034	1.472	0.0602	3	0.950	0.054	2.093	**0.0002**
			Treatment:Plant Species	9	1.322	0.061	0.886	0.7567	9	1.747	0.100	1.283	**0.008**
			Residuals	77	12.767	0.590			75	11.347	0.647		
			Total	92	21.647	1.000			90	17.542	1.000		
		rhizoplane	Treatment	3	5.652	0.235	9.163	**0.0001**	3	3.197	0.171	6.463	**0.0001**
			Plant Species	3	0.869	0.036	1.409	**0.0481**	3	1.016	0.054	2.054	**0.0001**
			Treatment:Plant Species	9	1.899	0.079	1.026	0.383	9	1.829	0.098	1.232	**0.0084**
			Residuals	76	15.628	0.650			77	12.698	0.678		
			Total	91	24.048	1.000			92	18.741	1.000		
		root	Treatment	3	4.742	0.176	6.490	**0.0001**	3	2.629	0.125	5.537	**0.0001**
			Plant Species	3	0.993	0.037	1.359	0.0532	3	4.138	**0.197**	8.716	**0.0001**
			Treatment:Plant Species	9	2.515	0.093	1.147	0.1094	9	1.865	0.089	1.309	**0.0036**
			Residuals	77	18.754	0.695			78	12.343	0.588		
			Total	92	27.003	1.000			93	20.974	1.000		

Supplementary Table 7

Effect of fertilization and plant species on the diazotroph and the general microbial community composition based on PERMANOVA using the Bray-Curtis dissimilarity index excluding samples from the NPK treatment. Significant effects are shown in bold.

				Diazotroph community					General microbial community				
Plants	Fertilization	Microenvironment	Factors	Df	SumsOfSqs	R^2	F	p-value	Df	SumsOfSqs	R^2	F	p-value
all	unfertilized, N, P	bulk soil	Treatment	2	6.618	0.388	20.700	**0.0001**	2	4.520	0.281	14.196	**0.0001**
			Plant Species	3	0.579	0.033	1.207	0.2213	3	0.860	0.053	1.801	**0.0080**
			Treatment:Plant Species	6	1.066	0.062	1.111	0.2769	6	1.257	0.078	1.316	0.0522
			Residuals	55	8.791	0.515			59	9.394	0.585		
			Total	66	17.054	1.000			70	16.032	1.000		
		rhizosphere soil	Treatment	2	5.388	0.330	16.255	**0.0001**	2	2.641	0.195	8.348	**0.0001**
			Plant Species	3	0.582	0.035	1.170	0.2292	3	0.920	0.068	1.940	**0.0005**
			Treatment:Plant Species	6	0.863	0.053	0.868	0.7040	6	1.216	0.090	1.281	**0.0262**
			Residuals	57	9.447	0.580			55	8.700	0.647		
			Total	68	16.282	1.000			66	13.478	1.000		
		rhizoplane	Treatment	2	4.3945	0.246	10.833	**0.0001**	2	2.375	0.165	6.933	**0.0001**
			Plant Species	3	0.7777	0.043	1.278	0.1178	3	1.008	0.070	1.962	**0.0001**
			Treatment:Plant Species	6	1.3129	0.073	1.079	0.2866	6	1.229	0.085	1.195	**0.0540**
			Residuals	56	11.3575	0.636			57	9.766	0.679		
			Total	67	17.8426	1.000			68	14.379	1.000		
		root	Treatment	2	3.3291	0.165	6.67	**0.0001**	2	1.970	0.123	6.102	**0.0001**
			Plant Species	3	0.9141	0.045	1.22	0.1378	3	3.584	**0.224**	7.398	**0.0001**
			Treatment:Plant Species	6	1.6513	0.082	1.103	0.2203	6	1.050	0.065	1.083	0.2482
			Residuals	57	14.2113	0.706			58	9.365	0.586		
			Total	68	20.1058	1.000			69	15.970	1.000		

Supplementary Table 8

Number (n) of investigated samples for (a) *nifH* and 16S rRNA gene sequencing of microenvironments associated with grasses and herbs across fertilization treatments, (b) sequencing expressed *nifH* sequences from cDNA samples of grasses, (c) abundance measurements via quantitative PCR (qPCR), (d) N_2 fixation potential analysis.

(a)

	Number (n) of investigated grass samples				Number (n) of investigated herb samples			
	bulk soil	rhizosphere	rhizoplane	root	bulk soil	rhizosphere	rhizoplane	root
unfertilized	12	12	12	12	12	12	12	12
N	12	12	12	12	11	11	11	11
P	12	12	12	11	12	12	12	12
NPK	12	12	12	12	12	12	12	12

(b)

	Number (n) of cDNA samples of investigated grasses			
	bulk soil	rhizosphere	rhizoplane	root
unfertilized	4	6	6	9

(c)

	Number (n) of investigated grass samples for qPCR		Number (n) of investigated herb samples for qPCR	
	bulk soil	rhizosphere	bulk soil	rhizosphere
unfertilized	11	11	11	12
N	11	12	11	11
P	11	11	12	12
NPK	11	12	12	10

(d)

	Number (n) of investigated samples for N_2 fixation potential analysis			
	Incubation days	unfertilized	N	P
Root exudates	3	3	3	3
	7	3	3	3
	21	3	3	3
no C addition	3	3	3	3
	7	3	3	3
	21	3	3	3
