# Supplementary material for: Plant roots affect free-living diazotroph communities in temperate grassland soils despite decades of fertilization
Source: Commun Biol. 2024 Jul 11;7:846. doi: 10.1038/s42003-024-06522-w (PMC11237082; doi:10.1038/s42003-024-06522-w)
Supplement: Supplementary file 3 — Description of additional supplementary files [file 42003_2024_6522_MOESM3_ESM.docx]

Description of Additional Supplementary Files

**File name:** Supplementary Data 1.1

**Description:** DeSeq2 results of differentially abundant diazotroph OTUs in microhabitat comparisons of individual field treatments associated to grasses.

**File name:** Supplementary Data 1.2

**Description:** DeSeq2 results of differentially abundant diazotroph OTUs in microhabitat comparisons of individual field treatments associated to herbs.

**File name:** Supplementary Data 1.3

**Description:** DeSeq2 results of differentially abundant diazotroph OTUs in pairwise field treatment comparisons of individual microhabitats of all plant species

**File name:** Supplementary Data 1.4

**Description:** Source data underlying Figure 3.

**File name:** Supplementary Data 1.5

**Description:** Source data underlying Figure 6.
